# Allergic hemiglossitis as a unique case of food allergy: a case report

**DOI:** 10.1186/1752-1947-2-71

**Published:** 2008-03-06

**Authors:** Omar Aziz, Csaba Dioszeghy

**Affiliations:** 1 Accident and Emergency Department, Yeovil District Hospital, Higher Kingston, Yeovil, Somerset BA21 4AT, UK

## Abstract

**Introduction:**

A unique case of topical food allergy is presented with unilateral swelling of the tongue with ulceration. Only one similar case has been reported in 1972.

**Case presentation:**

76 years old female patient presented at the Emergency Department with a unilateral painful swelling and ulcerations of the tongue after eating mint chocolate. However the swelling did not compromised the airways the presentation was rather frightening for the patient. The allergic reaction responded to the parenteral steroid and oral chlorphenamine treatment and the three month follow up only revealed minimal scar formations.

**Conclusion:**

Unilateral hemiglossitis is a rare form of allergic reactions which is usually self-limiting with full recovery of which takes an unusually long time.

## Introduction

Allergic reactions often cause swelling in the tongue, which is usually more frightening than dangerous. Hypersensitivity to specific food or medications is believed to be the most common cause [1-3]. However, the airway might become compromised and life-threatening manifestations have also been reported [4]; therefore, these patients are relatively aggressively treated and observed for as long as necessary. These allergic reactions are usually more diffuse, often with swelling of the glottis and are presented along with other systemic symptoms.

Localised swelling of only one side of the tongue was described by Chavanne as allergic hemiglossitis in 1972, and was related to alimental allergic reaction [5]. He also noted that the swelling was accompanied by the development of ulcers and the recovery took several months.

## Case presentation

A 76-year-old otherwise fit and healthy female presented at the Accident and Emergency Department at 2:15 am with the complaint of a mildly painful and remarkably swollen tongue that developed after eating mint chocolate. Although the patient had tenderness and the frightening swelling in the mouth causing discomfort and moderate difficulty in swallowing, she had no shortness of breath or any other complaint. She gave a medical history of rheumatic polimyalgia in the past requiring no medical treatment at present. She remembered a previous allergic reaction to mint.

**Figure 1:**
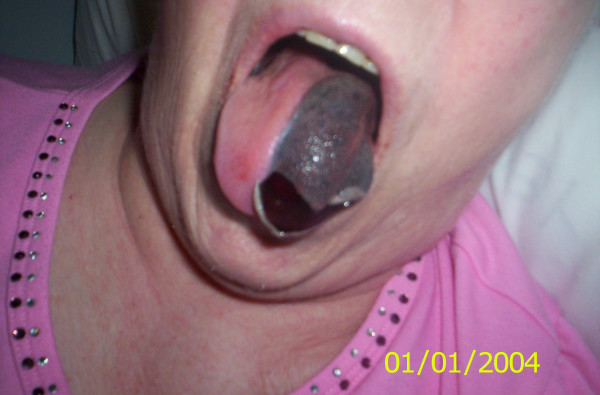
Localised swelling on the left-hand side of the tongue.

On examination it was noted that a remarkable swelling was localised only on the lefthand side of the tongue (please see Figure 1). The tongue seemed to be suffused and a superficial non-tender ulceration was seen at the anterior quarter of the surface. There was a mild tenderness and a loss of taste on this side. The right-hand side of the tongue was completely normal. Tongue movement was normal apart from mild restriction caused by the swelling. No other pathology on the oral mucosa or in the throat was observed.

There was no skin rash or any other systemic reaction, and the vital parameters were stable. Blood results showed a mild elevation of C-reactive protein (CRP = 17) and erythrocyte sedimentation rate (ESR = 33).

As the symptoms were considered as a local allergic reaction, the patient was given 4 mg chlorphenamine orally and 100 mg hydrocortisone injection intramuscularly. The swelling responded to the medication quickly and the patient was discharged after a period of observation.

**Figure 2:**
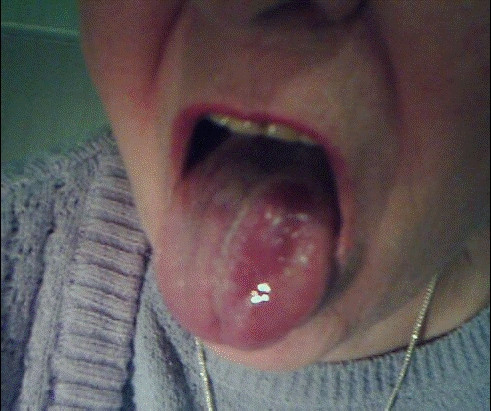
A scar is still visible three months later.

We made a follow up after one and three months. However, although the swelling had almost completely gone by the next morning and the tongue had become pain free, the follow up revealed that the healing process was indeed longer. Even after three months a scar was seen on the top of the left-hand side of the tongue (please see Figure 2). No other disability was reported and the sense of taste had also returned to normal.

## Discussion

The presented case is a rare, usually benign, but quite frightening manifestation of a food allergy which is typically a Type I allergic reaction developing within seconds or minutes after contact with the allergen. Our patient had a similar initial presentation to the only other known case reported in [5] and had a similarly slow resolution.

However, allergic glossitis is described as a self-limiting condition with a full recovery; the hemiglossitis form we have presented proved to be slower healing with some scarring still visible after three months. Nevertheless, this caused no complaint for the patient. Clinicians should be aware of this in order to give reassuring information to their patients.

## Conclusion

Allergic hemiglossitis is a unique form of localised allergic reaction most likely caused by food. The swelling responds promptly to anti-allergic medication but the ulceration takes longer to heal. Recovery may take up to three months.

## Competing interests

The author(s) declare that they have no competing interests.

## Authors' contributions

OA examined the patient, provided accurate management and arranged the initial case presentation report. CD provided the references and amended the presentation.

## Consent

Written informed consent was obtained from the patient for publication of this case report and accompanying images. A copy of the written consent is available for review by the Editor-in-Chief of this journal.
